# Comparing Traditional Versus AI‐Assisted TMJ Disorder Management Approaches: A Systematic Review and Meta‐Analysis

**DOI:** 10.1002/cre2.70370

**Published:** 2026-04-28

**Authors:** Vini Mehta, Annie Vathani, Praveen Kumar Gonuguntla Kamma, Saurabh Chaturvedi, Ankita Mathur, Toufiq Noor

**Affiliations:** ^1^ Dental Research Cell, Dr. D. Y. Patil Dental College & Hospital, Dr. D. Y. Patil Vidyapeeth (Deemed to be University) Pune India; ^2^ Faculty of Dentistry University of Ibn al‐Nafis for Medical Sciences Sana'a Yemen; ^3^ Kalam Institute of Health Technology Visakhapatnam Andhra Pradesh India; ^4^ Celebrate Dental & Braces Walzem Road San Antonio Texas USA; ^5^ Department of Prosthetic Dentistry, College of Dentistry King Khalid University Abha Saudi Arabia

**Keywords:** artificial intelligence, diagnostic accuracy, machine learning, meta‐analysis, radiomics, systematic review, temporomandibular joint disorder

## Abstract

**Objective:**

This systematic review and meta‐analysis compared traditional diagnostic approaches with artificial intelligence (AI)‐based techniques for temporomandibular joint disorders (TMDs) and evaluated their diagnostic accuracy.

**Methods:**

Literature searches across PubMed‐MEDLINE, Scopus, Embase, Cochrane, and Google Scholar identified studies published between 1st January 2010 to 20th April 2025. Eligible studies used AI‐based algorithms, such as deep learning (DL), machine learning (ML), or computer‐aided diagnostic tools, for TMD diagnosis or management, reporting performance metrics including sensitivity, specificity, and accuracy. Traditional approaches, including clinical examinations, radiographic assessments, and standardized diagnostic criteria (DC/TMD), serve as comparators. Risk of bias was assessed using Quality Assessment of Diagnostic Accuracy Studies (QUADAS‐2). Meta‐analysis was conducted in the form of pooled sensitivity and specificity.

**Results:**

Fourteen studies were included, comprising AI models trained on clinical and imaging data including cone beam computed tomography (CBCT), magnetic resonance imaging (MRI), and orthopantomogram (OPG). AI methods showed moderate‐to‐high diagnostic accuracy, with sensitivity ranging from 0.66 to 0.88 and specificity from 0.72 to 0.86. A meta‐analysis of five studies showed pooled sensitivity and specificity estimates within these ranges. Among the included studies, AI models integrated radiomic as well as semantic features to achieve sensitivity from 0.82 to 0.93, and specificity from 0.76 to 0.90; however, evidence showed low certainty because bias risk was high (7/9 studies), sample sizes were small (mean *n* = 42), and external validation was absent in 8 of 9 studies.

**Conclusion:**

AI‐assisted techniques offer significant potential to complement traditional TMD diagnostic methods by enhancing the diagnostic precision. However, owing to methodological limitations, further high‐quality prospective studies with standardized reporting are needed to validate the use of AI in TMD management.

## 1 Introduction

Temporomandibular disorders (TMDs) represent a group of conditions that affect the temporomandibular joint (TMJ), the masticatory muscles, and related anatomical structures. These conditions often lead to pain, functional limitations, and joint sounds, contributing significantly to nonodontogenic orofacial pain. The prevalence of TMD is estimated to range from 3.7% to 12%, with a higher occurrence observed in women. TMD not only affects physical health but also has a considerable impact on psychological well‐being, often coexisting with conditions such as depression. It also contributes to socioeconomic burden due to reduced work productivity and increased healthcare utilization (Shoukri et al. 2019). Over the decades, the understanding and management of TMD have evolved considerably. Prior to the 1980s, malocclusion was widely believed to be the primary cause of TMD. However, by the 1990s, this theory was largely refuted, and orthodontic treatments were no longer recommended as standard care. Between 2000 and 2010, the focus shifted toward invasive procedures such as arthrocentesis and arthroscopy. Despite initial interest, subsequent evidence demonstrated limited clinical efficacy for these approaches. Since 2010, there has been a growing emphasis on the biopsychosocial model of TMD, supported by advancements in neuroscience. This model considers the complex interplay between physiological, emotional, and psychological factors, and has led to the adoption of more holistic, evidence‐based treatment strategies (Gauer and Semidey 2015; Gil‐Martínez et al. 2018).

TMDs are multifactorial in nature, involving dysfunction of the TMJ, associated musculature, and surrounding tissues (Jha et al. 2022). Accurate diagnosis often requires a comprehensive evaluation that integrates clinical examination, imaging findings, and assessment of behavioral and psychosocial variables. However, due to the heterogeneous presentation of TMD, diagnosis remains challenging in routine clinical practice (Almășan et al. 2023). The Diagnostic Criteria for Temporomandibular Disorders (DC/TMD) is the most widely accepted classification system for TMD diagnosis. It consists of two axes; Axis I categorizes pain‐related and intra‐articular disorders, while Axis II evaluates jaw function and psychosocial factors. Although the DC/TMD has contributed to standardizing TMD diagnosis globally, it has limitations. Its sensitivity and specificity for certain conditions, such as disc displacement and degenerative joint disease (DJD) range from 0.34 to 0.61, and inter‐examiner reliability remains variable, limiting its diagnostic robustness (Schiffman et al. 2014). These limitations highlight the need for adjunctive diagnostic tools that can enhance accuracy and reproducibility.

In recent years, artificial intelligence (AI) has emerged as a transformative tool in healthcare, with growing applications in diagnostic support. By analyzing large and complex datasets, AI systems have demonstrated potential in pattern recognition and disease prediction across various specialties, including dermatology, ophthalmology, oncology, and dentistry (de Dumast, Mirabel, Cevidanes et al. 2018). In the context of TMD, AI particularly using medical imaging and structured clinical data has been explored to support diagnosis and classification (Choi et al. 2021). AI encompasses a range of computational approaches designed to perform tasks traditionally associated with human cognition. Within AI, machine learning (ML) refers to techniques that enable systems to learn patterns from data and make predictions without being explicitly programmed. Deep learning (DL), a subset of ML, uses layered neural networks to process complex datasets and has shown strong performance in tasks involving image recognition and classification. In dentistry, DL has become increasingly relevant for analyzing diagnostic imaging, including radiographs and 3D scans. Its ability to extract meaningful features from multimodal data, including images, sensor outputs, and clinical text, makes it particularly well‐suited for use in medical diagnostics (Lee et al. 2022; Mao et al. 2025; Xu et al. 2023).

Unlike many other medical specialties, dental diagnostics are frequently performed chairside and depend on operator‐controlled imaging acquisition and clinician interpretation, which can introduce variability in image quality and diagnostic reproducibility (Schiffman et al. 2014). Differences in CBCT positioning, artifacts, and manual annotations may influence downstream AI model training and performance. Moreover, because dental decision‐making directly informs immediate clinical interventions, medico‐legal accountability and clinician trust require transparent and interpretable AI outputs rather than “black‐box” predictions (Jha et al. 2022; de Dumast, Mirabel, Cevidanes et al. 2018; Choi et al. 2021). Consequently, concerns regarding explainability, bias, and generalizability are particularly salient in dental AI applications, underscoring the need to critically evaluate the trustworthiness of such systems within routine TMD workflows.

Conventional diagnostic approaches for TMD rely on clinical history, physical examination, and radiographic interpretation. However, the emergence of AI‐powered diagnostic tools and computer‐aided diagnosis (CAD) systems leveraging ML and DL techniques offers new opportunities to enhance diagnostic precision and support clinical decision‐making. These technologies have been applied to TMD assessment using various data modalities, including cone‐beam computed tomography (CBCT), panoramic radiography, magnetic resonance imaging (MRI), and clinical symptom profiles. Nevertheless, existing studies vary widely in terms of population characteristics, methodologies, and outcome measures. Therefore, the aim of this review is to compare AI‐based diagnostic methods with conventional clinical approaches in the diagnosis and management of temporomandibular joint disorders. This review seeks to provide a comprehensive evaluation of the potential for AI to augment traditional diagnostic frameworks and inform the future of clinical practice in TMD care.

## 2 Materials and Methods

The systematic review and meta‐analysis were carried out and documented following the PRISMA 2020 guidelines for systematic reviews and meta‐analyses (Page et al. 2021) (Table S1). The protocol for this review was registered with PROSPERO, registration ID: CRD420251036138.

### 2.1 Review Question

What are the comparative outcomes of traditional versus AI‐assisted approaches in the diagnosis and management of TMJ disorders?

### 2.2 PICOT Framework

∘ **P (Population):** Patients diagnosed with TMDs.
∘ **I (Intervention):** AI‐assisted management approaches.
∘ **C (Comparison):** Traditional management approaches.
∘ **O (Outcomes):** Diagnostic accuracy, treatment outcomes, efficiency, safety.
∘ **T (Type of Study):** Comparative studies, diagnostic accuracy studies, observational studies.

### 2.3 Search Strategy

A detailed search was developed to identify studies on using artificial intelligence (AI) for diagnosing and managing temporomandibular joint (TMJ) disorders. This search was carried out using online databases and gray literatures such as PubMed, Scopus, Embase, Web of Science Database, and Google Scholar from 1st January 2010 to 20th April 2025. Only studies in the English language were included. First, a specific search plan was made for MEDLINE (PubMed) using Medical Subject Headings (MeSH) and keywords about TMJ disorders and AI, linked with Boolean operators. For the gray literature, we included only 20 pages of the website. If there is missing literature, authors have been contacted twice. This strategy was then adapted to suit the syntax and indexing terms of the other databases. A detailed search strategy was given in Table S2.

### 2.4 Eligibility Criteria and Study Selection

This systematic review included original research articles published in peer‐reviewed journals. Eligible studies employed artificial AI algorithms such as ML, DL, or CAD for the diagnosis, classification, or management of at least one subtype of TMDs. Studies were required to evaluate AI model performance using metrics like sensitivity, specificity, accuracy, and to compare AI‐assisted methods with traditional diagnostic or management techniques (e.g., clinical examination based on RDC/TMD or DC/TMD criteria, conventional imaging, or standard therapeutic approaches) (Schiffman et al. 2014).

There were no restrictions on participant age, sex, or ethnicity. Studies were excluded if they focused on orofacial pain not directly related to TMDs (e.g., atypical facial or neuropathic pain), examined TMJ anatomy or biomechanics without diagnostic or therapeutic relevance, lacked empirical data (e.g., editorials, commentaries, book chapters, conference abstracts), or did not report AI algorithm performance.

### 2.5 Study Selection and Data Extraction

After database searches, duplicate entries were removed using Zotero, with manual verification by one researcher. Titles and abstracts were screened independently by two reviewers (A.N.V.J. and S.C.) using predefined eligibility criteria. Full texts of potentially relevant studies were assessed, and disagreements on inclusion were resolved through discussion with a third reviewer (V.M.). Data extraction was independently performed by the two reviewers using a standardized Excel sheet. Extracted information included study sample characteristics (e.g., country, age, sex), type of AI algorithm used, input data, diagnostic or management approach, details of control groups, follow‐up, and outcomes.

### 2.6 Categorization of Explainability and Interpretability Approaches

As the heterogeneity of interpretability techniques reported across studies (e.g., Grad‐CAM, SHAP, LIME, rule‐based systems), explainability evidence was synthesized qualitatively rather than numerically. Methods were grouped a priori into conceptually coherent domains, including saliency‐based visualization, feature‐attribution approaches, and rule‐ or model‐inherent interpretability. Classification was based on the reported purpose, level of explanation (local vs. global), and potential clinical interpretability, enabling structured comparison across studies while preserving methodological differences. Concordance between reviewers was verified, and discrepancies were resolved by consensus with input from the third reviewer when needed.

### 2.7 Risk of Bias Assessment of the Included Studies

Two reviewers (A.N.V.J. and S.C.) independently and in duplicate evaluated the risk of bias and applicability concerns in the primary diagnostic accuracy studies included in the analysis, utilizing the QUADAS‐2 (Quality Assessment of Diagnostic Accuracy Studies 2) tool (Whiting et al. 2011). Each reviewer assessed all included studies separately to ensure independent judgment and minimize subjective bias. This tool consists of four main domains: Patient Selection, Index Test, Reference Standard, and Flow and Timing. Each domain is scrutinized for bias risk, while the first three domains are also assessed for applicability concerns. Responses to each question are classified as “Yes,” “No,” or “Unclear.” Based on these responses, each domain is rated as having a “Low,” “High,” or “Unclear” risk of bias and applicability concern. The overall assessment of a study's bias risk is not aggregated across domains but is instead described in detail, with explanations provided for each judgment. Any disagreements between reviewers were first resolved through discussion and consensus; if consensus could not be reached, arbitration was performed by a third reviewer (V.M.). This standardized independent assessment and adjudication process was implemented to enhance methodological transparency, reliability, and reproducibility of the qualitative synthesis.

To assess potential publication bias, we generated a funnel plot of the diagnostic odds ratios (DOR) against their standard errors for the included studies. Visual asymmetry in the funnel plot may indicate small‐study effects or publication bias. Egger's regression test was also conducted to provide a statistical evaluation of funnel plot asymmetry. A *p*‐value < 0.05 was considered to suggest significant bias. We note that the small number of included studies (< 10) limits the power of these tests.

### 2.8 Statistical Analysis

Meta‐analysis of diagnostic accuracy was conducted using Review Manager (RevMan v5.4, Cochrane Collaboration). Pooled sensitivity and specificity were calculated. Heterogeneity across studies was assessed using the I² statistic. As the anticipated clinical and methodological heterogeneity across studies (e.g., differences in imaging modalities, AI architectures, sample sizes, and outcome reporting), studies were not assumed to be directly comparable. Accordingly, pooled analyses were intended to summarize overall trends rather than imply causal or performance superiority, and findings were interpreted cautiously within the context of task type and data characteristics. Where applicable, subgroup analyses were conducted to compare performance between AI‐based tools and conventional approaches. All diagnostic performance metrics were derived from 2 × 2 contingency tables (true positives, false positives, true negatives, and false negatives). RevMan's built‐in Mantel–Haenszel method was used under a random‐effects model to account for between‐study variability. Although five studies were identified and evaluated for diagnostic effectiveness, not all could be included in the pooled meta‐analysis. Certain studies were excluded due to insufficient or incompatible data reporting, for example, some did not provide the full set of diagnostic metrics (e.g., sensitivity, specificity, or 2 × 2 contingency tables) necessary to compute pooled estimates.

Others used non‐standard reference criteria or outcome measures that were not comparable with most included studies, which would have introduced bias and reduced the validity of the pooled results.

To assess the robustness of the pooled diagnostic performance estimates, a leave‐one‐out (LOO) sensitivity analysis was performed. The changes in these metrics compared with the overall pooled estimates were summarized in tabular form and visualized using bar charts. This analysis allows identification of studies exerting disproportionate influence on the pooled results.

We fitted a Reitsma bivariate random‐effects model (R, version 4.4.1; **mada** package, version 0.5.11) using 2 × 2 tables (TP, FN, FP, TN) from each study. The summary ROC (sROC) curve was plotted with study points, a summary point (pooled sensitivity and specificity with 95% CI ellipse), and a 95% prediction ellipse. Model estimates were reported as pooled sensitivity, specificity, area under the curve (AUC), and partial AUC (pAUC) restricted to observed false‐positive rates.

#### 2.8.1 Binary Coding of Qualitative Study Characteristics

To enable structured quantitative comparison across heterogeneous studies, selected qualitative methodological attributes (e.g., explainability reporting, fairness assessment, external validation, and bias mitigation) were dichotomized using predefined criteria. The explicit presence of a feature was coded as “1” and absence or unclear reporting as “0.” Coding was performed independently by two reviewers with consensus resolution of discrepancies. While this pragmatic approach facilitated uniform comparison, binary categorization may oversimplify nuanced methodological differences, and findings were interpreted cautiously.

#### 2.8.2 Exploratory Correlation Analysis

To explore potential associations between study characteristics (e.g., reporting of explainability, fairness, validation practices) and model performance outcomes, selected qualitative attributes were dichotomized (presence/absence) to enable structured comparison across heterogeneous studies. As the variability in study designs, outcome measures, and reporting standards, this binary coding approach was adopted for pragmatic synthesis but may oversimplify nuanced methodological differences. Therefore, correlation analyses were conducted strictly as exploratory assessments to identify descriptive patterns rather than to establish causal or inferential relationships. Results were interpreted cautiously in light of the small number of included studies and methodological heterogeneity, and statistical significance was not considered confirmatory.

## 3 Results

### 3.1 Study Selection

**Figure 1:**
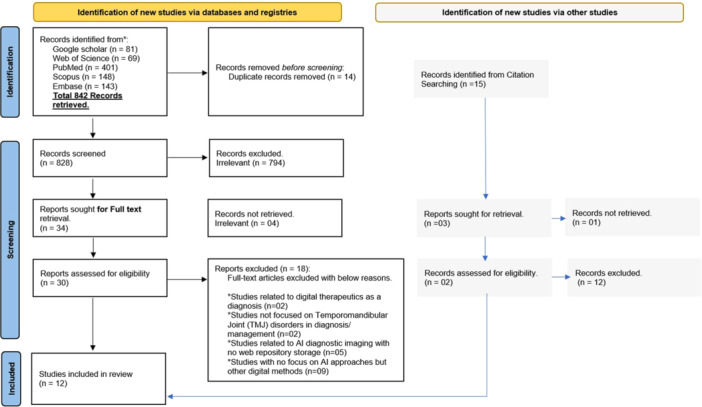
PRISMA flow diagram.

The initial search strategy yielded 842 articles from various databases, and 14 duplicates were removed. After a full‐text review, 13 studies were selected for inclusion (Shoukri et al. 2019; de Dumast, Mirabel, Cevidanes et al. 2018; Choi et al. 2021; Lee et al. 2022; Talaat et al. 2023; Ito et al. 2022; Haghnegahdar et al. 2018; Orhan et al. 2021; Bianchi et al. 2020; Ribera et al. 2019; Mackie et al. 2022; Al Turkestani et al. 2023; Diniz de Lima et al. 2022). Excluded studies were basically from the deviation of the expected outcome, which cannot be assessed (Figure 1). The full list of excluded studies is given in Table S3.

### 3.2 Characteristics of the Study

These studies, published between 2018 and 2023, encompassed a range of geographic locations and methodologies, highlighting a growing global interest in utilizing AI, ML, and DL techniques for diagnosing and managing TMJ disorders. The sample sizes varied significantly between 20 and 3514 patients or imaging records. Most participants were adults experiencing TMJ‐related symptoms, with a higher prevalence of females in several studies, which is consistent with the known epidemiological trends of TMJ disorders. A subgroup comparison between adults, adolescents, and children was not feasible because most studies did not report age‐stratified outcomes, predominantly included adult populations, and demonstrated inconsistent age reporting formats, limiting meaningful categorization and pooled analysis. Various data sources were employed, including panoramic radiographs (OPG), CBCT, MRI, and clinical symptom profiles. Radiomic feature extraction methods, such as Local Binary Patterns (LBP), Histogram of Oriented Gradients (HOG), and texture analysis, are frequently used to enhance diagnostic input from imaging modalities. The AI methodologies applied across the studies included both traditional ML classifiers (e.g., K‐Nearest Neighbors, Support Vector Machines, Logistic Regression, Decision Trees, Random Forest) and advanced DL models (e.g., Convolutional Neural Networks, ResNet, U‐Net, SegNet‐Basic, Single‐Shot Detectors, and 3DiscNet). Performance evaluation typically involves comparison with traditional diagnostic standards, such as clinical examination based on RDC/TMD or DC/TMD criteria or manual interpretation by radiologists. Outcome measures varied but generally included sensitivity, specificity, accuracy, area under the curve (AUC), precision, recall, and the Dice similarity coefficient for segmentation tasks. Studies generally reported favorable or comparable predictive performance across both traditional machine learning and deep learning approaches relative to expert‐driven diagnoses. However, results were variable and dependent on task type, data modality, and study design, rather than indicating a consistent advantage of any specific model class, and should therefore be interpreted descriptively given methodological heterogeneity (Table 1).

**Table 1:** Characteristics of included studies (*n* = 14).

Study ID	Condition	Country	Study design	Sample size	Age group (in years)	Population characteristics	AI techniques used	Comparators	Methods	Outcome
Bianchi et al. (2020)	TMJ OA	NR	Diagnostic study design	92 subjects, 46 in each group (TMJ OA patients and controls)	21–70 years	Non‐invasive protocol used to detect the initial morphological changes in the mandibular condyle trabecular bone based on radiomics information between patients and control groups	Logistic Regression, Random Forest, LightGBM, and XGBoost. These models were used to analyze various clinical and biological markers	Traditional diagnostic methods using clinical examination using Research Diagnostic Criteria (RDC/TMD) and radiographic findings using x‐ray or MRI	52 clinical, biological, and high‐resolution CBCT (radiomics) markers were analyzed. Area under the curve (AUC) were evaluated.	AI methods such as XGBoost + LightGBM model achieves the accuracy = 0.823, AUC = 0.870, and F1‐score 0.823
Choi et al. (2021)	TMJ OA	Seoul National University Dental Hospital between January 2015 to October, 2019	Comparative study design	1189 OPG images	≥ 18 years (upper age not reported)	Patients who visited the orofacial pain clinic who reported TMD‐related symptoms were included	AI Keras' ResNet model, a type of Convolutional Neural Network (CNN).	Oromaxillofacial radiology (OMFR) expert with a criterion (research and diagnostic criteria for TMD14, TMD 15)	The study involved the (ROIs) from OPG images, focusing on the mandibular condyle, articular fossa, and eminence.	AI model achieved a balance in diagnostic performance, with an accuracy = 0.78, sensitivity = 0.73, specificity = 0.82, demonstrating its potential role in primary diagnosis of TMJOA
de Dumast, Mirabel, Cevidanes (2018)	TMJ OA	University of Michigan	Cross‐sectional clinical study	259 condyles, with 105 from control subjects and 154 from patients diagnosed with TMJ OA.	Mean age = 39.9 ± 11.7 (patients); 39.4 ± 15.4 (controls)	Individuals with degrees of TMJ OA, as well as control subjects who did not exhibit any signs or symptoms of OA. Average age = 39.4 years, with a SD = 15.4 years	Shape variation analyzer (SVA) to analyze 3D condylar morphology.	Clinician consensus on the classification of condylar morphology, which served as the gold standard	The study incorporated Multivariate Functional Shape Data Analysis (MFSDA) and Principal Component Analysis (PCA) to analyze the relationships between 3D mesh data and various clinical, demographic, and biological variables	The outcomes of the study demonstrated a high level of agreement (91%) between the neural network classifier and clinician consensus
Haghnegahdar et al. (2018)	Temporomandibular joint disorder (TMD)	Dentistry Department of Shiraz University, Iran.	Diagnostic study design	Healthy individuals = 132 images Patients with TMD, = 132 images Total = 264 images	Not‐reported	66 patients with TMD and 66 normal cases, with images specifically focused on the head of the mandibular condyle	Local Binary Patterns (LBP), Histogram of Oriented Gradients (HOG) and K‐Nearest Neighbor (K‐NN)	The study compared the performance of the K‐NN classifier against other classifiers, including **Support Vector Machine (SVM)**, **Naïve Bayesian**, and **Random Forest**	The study utilized (CBCT) images for analysis. Feature extraction, texture and structural patterns were performed. ROC curves were evaluated for performance of classification model.	K‐NN classifier, which achieved an accuracy= 92.42%, sensitivity= **94.70%**, and specificity = **90.15%**
Ito et al. (2022)	Temporomandibular joint (TMJ) disorders, specifically focusing on articular disc displacement and deformation	Hiroshima University, Japan	Non‐randomized retrospective study	217 MRI images, including a total of 20 subjects 10 patients= anterior displacement of the articular disc and 10 = healthy control subjects	19–39 years (mean = 26.4 years)	The patients with articular disc displacement were aged 19 to 39 years (mean age of 26.4; 8 women, 2 men).	Deep learning‐based semantic segmentation algorithms, specifically: 3DiscNet, SegNet‐Basic and U‐Net	**Manual segmentation data** performed by experts	The study utilized **MRI images** for segmentation. The evaluation metrics included the **Dice coefficient**, **sensitivity**, and **positive predictive value (PPV)** for both groups.	**SegNet‐Basic:** Dice: 0.74 Sensitivity: 0.70 PPV: 0.80 **U‐Net:** Dice: 0.46 Sensitivity: 0.44 PPV: 0.54 3DiscNet: Dice: 0.70 Sensitivity: 0.60 PPV: 0.80
Kim et al. (2020)	TMJ OA	AIQUB dental network in South Korea	Retrospective study design	1292 patients. Men = 700 Women = 592	20–60 years	The patient population consisted of individuals who had undergone treatment aged between 20 and 60 years	Convolutional Neural Networks (CNNs) Faster Region‐based Convolutional Networks (R‐CNNs)	Traditional diagnostic methods	This study involved a retrospective evaluation of panoramic radiographic and a two‐stage deep learning approach was used.	Sensitivity = 0.54 Specificity = 0.94 and accuracy = 0.84
Lee et al. (2022)	TMJ OA	Korea University Anam Hospital	Diagnostic tool development design	3514 CBCT images derived from 314 patients	16–84 years (mean = 39.5 years)	The population included 84 males and 230 females, with a mean age of 39.5 years (age range: 16–84 years).	Single‐shot detector (SSD), a deep learning framework	Two test sets of 300 image	**Accuracy, precision, recall, and F1 score** metrics were calculated based on the intersection‐over‐union (IOU) threshold of **0.50**.	The study found that automated detection of TMJOA from sagittal CBCT images was feasible, achieving an average accuracy = 0.86 precision = 0.85 recall = 0.84
Diniz de Lima et al. (2022)	Temporomandibular disorder (TMD)	Brazil	Cross‐sectional observational diagnostic study	78 subjects (41 TMD patients, 37 controls)	18–60 years	Adults aged 18–60 years; matched for age, sex, and BMI; patients recruited from Orofacial Pain Clinic; RDC/TMD and Fonseca questionnaire used for classification	K‐Nearest Neighbors (KNN), Support Vector Machine (SVM), and Multilayer Perceptron (MLP) applied on three feature extraction methods: radiomic, semantic, and radiomic‐semantic association	Clinical classification using RDC/TMD and Fonseca questionnaire; thermographic imaging evaluated by radiologist	Infrared thermography (IT) images of masseter and temporalis muscles acquired and processed to extract texture (radiomic) and thermal/pain score (semantic) features; 156 images analyzed; statistical validation using ANOVA, Tukey, Shapiro–Wilk, and Hopkins's statistic	Radiomic‐semantic association using MLP classifier showed highest accuracy; semantic and radiomic‐semantic methods outperformed pure radiomic features (*p* < 0.05); AI + IT promising for TMD detection
Mackie et al. (2022)	TMJ OA	University of Michigan	prospective study design	92 patients, TMJ OA = 46 patients, and control subjects = 46 resulting in 184 h‐CBCT scans	21–70 years	21–70 years of age included with year no history of systemic disease, history of TMJ trauma, surgery, or recent TMJ injections.	TMJOAI (TMJ Osteoarthritis Artificial Intelligence TMJPI (TMJ Privileged Information) ‐ Learning Using Privileged Information (LUPI)	Traditional clinical assessments and imaging features to evaluate their effectiveness in diagnosing TMJ OA	The study involved the use of h‐CBCT scans for radiographic interpretation by oral and maxillofacial radiologists. Machine learning models were trained using various features, including clinical, radiomic, and biological data.	The primary outcome was the diagnostic performance of the machine learning models in detecting TMJ OA status. Light GBM model AUC = 0.842.
Orhan et al. (2021)	Temporomandibular joint (TMJ) disorders	NR	Retrospective cohort study	Total= 214 TMJs 107 patients, Males= 34, females = 73	19–74 years (mean = 38 ± 17.97)	The participants were selected based on specific inclusion criteria, which included patients with anterior disc displacement with and without reduction.	K‐nearest neighbors (KNN), random forest (RF), logistic regression (LR), decision tree (DT), XGBoost, and SVM for feature selection, classification, and prediction of TMJ pathologies	Traditional methods using MRI	The study utilized a radiomics platform to extract imaging features from MRIs and AI models which included first‐order statistics, shape, texture, and various gray‐level matrices.	Sensitivity = 1.00 and AUC = 0.89 for the training set. Specificity = 0.74 and AUC = 0.77 for the testing set.
Ribera et al. (2019)	TMJOA	NR	NR	293 TMJ CBCT	Mean age = 39.9 ± 11.7 years	The dataset comprised 3D meshes classified into six groups by two expert clinicians, indicating a diverse representation of TMJ condition	The primary AI technique employed in this study is a deep learning neural network.	ML algorithms, including Support Vector Machines (SVM), Gaussian Process, and Random Forest	The neural network was trained for 100 epochs with a batch size of 32, using a learning rate set to 1–5. The training process involved combining geometric features with a shape descriptor known as the heat kernel signature to enhance classification accuracy	SVA is a promising tool for classifying TMJ osteoarthritis based on 3D morphology.
Shoukri et al. (2019)	Temporomandibular joint osteoarthritis	NR	comparative study design	34 participants in total: 17 TMJOA patients and 17 age‐ and sex‐matched control subjects.	Mean age = 39.9 ± 11.7 (patients); 39.4 ± 15.2 (controls)	The patients had been experiencing symptoms for less than 10 years. The average age of TMJOA patients was 39.9 years (±11.7), while the control group had an average age of 39.4 years (±15.2).	NN trained to classify different degrees of shape deformation in TMJOA condyles	Classifications made by two expert clinicians, who assessed the condylar morphology	Clinical examinations included questionnaires, visual analog scales for pain, and measurements of mouth opening. collected blood and saliva samples to quantify inflammatory biomarkers using protein microarrays. High‐resolution CBCT scans performed to analyze condylar morphology	The NN demonstrated predictive analytics with 73.5% and 91.2% accuracy when compared to the clinicians' consensus classification
Talaat et al. (2023)	TMJ OA	Sharjah, United Arab Emirates, between November 2020 and November 2022	Retrospective study	A total of 2737 CBCT from 943 patients with 792 osteoarthritic joints	18–80 years	Patients aged between 18 to 80 years who visited the clinic	AI model from CBCT images through convolutional network	Radiologists who traditionally interpret the CBCT images.	Cohen's kappa analysis	Assessing the accuracy and agreement of the AI model relative to the radiologists' interpretations which includes Diagnostic Performance, Statistical Agreement and Reduction of Subjectivity
Al Turkestani et al. (2023)	TMJ OA	University of Michigan School of Dentistry.	Diagnostic model development and validation study	46 patients + 46 controls	Not‐reported	Subjects diagnosed with TMJ OA 46 age and gender‐matched healthy controls.	LUPI paradigm	Clinical features alone and combinations of clinical and radiomic features	Utilizing CBCT scans for radiomics analysis focused on specific anatomical regions prone to OA degeneration	The study achieved an AUC = 0.81, specificity = 0.79, and precision = 0.77, indicating a robust performance in diagnosing TMJ OA

### 3.3 Risk of Bias Assessment of the Included Studies

**Figure 2:**
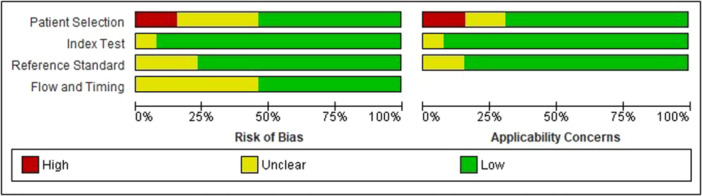
Risk of bias and applicability concerns graph.

**Figure 3:**
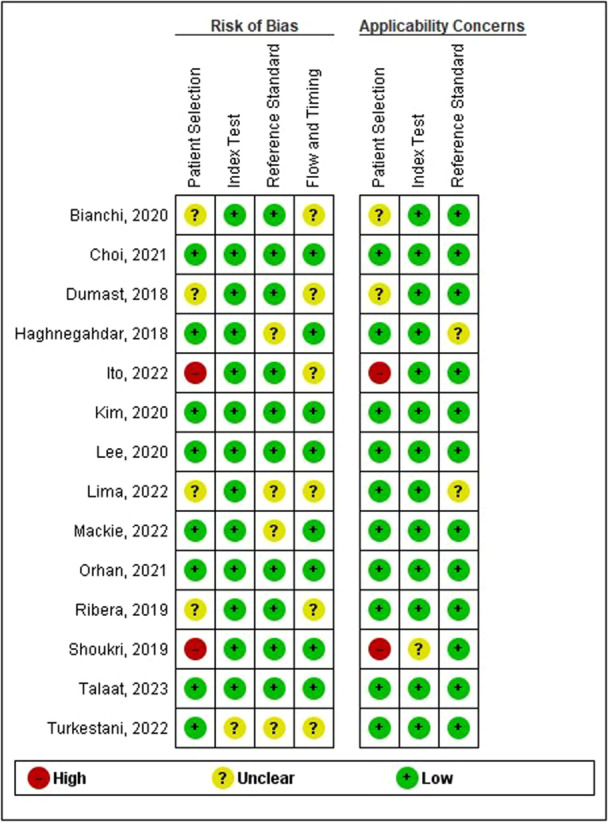
Risk of bias and applicability concerns summary.

The potential for bias and concerns about applicability in the studies included were evaluated using the QUADAS‐2 tool, as depicted in Figures 2 and 3. In the patient selection category, the majority of studies (10 out of 13) exhibited a low risk of bias. However, three studies (Shoukri et al. 2019; Ito et al. 2022; Haghnegahdar et al. 2018) were identified as having either a high or uncertain risk, primarily due to the use of cross‐sectional and case‐control designs or insufficient reporting of the sampling method. Nevertheless, most studies maintained low applicability concerns regarding patient selection, with only two studies (Shoukri et al. 2019; Bianchi et al. 2020) being rated as high or unclear due to less representative populations. All studies were found to have a low risk of bias in the index test category. Similarly, the reference standard category showed a low risk across all studies, although applicability concerns were unclear for the study (Haghnegahdar et al. 2018) due to a lack of detailed information on the alignment of the reference test. The flow and timing categories consistently received low‐risk ratings in all included studies, indicating appropriate timing and comprehensive data inclusion. Overall, most studies demonstrated low risk and low applicability concerns across key categories, which provides contextual information for interpretation but does not eliminate the impact of heterogeneity or small study sizes on the overall certainty of evidence.

### 3.4 Publication Bias

The funnel plot of the five included studies appeared broadly symmetrical around the pooled effect size, with no obvious visual indication of publication bias; however, interpretation remains limited due to the small number of studies. Egger's regression test did not detect significant asymmetry (*p* = 0.377). However, given that fewer than 10 studies were available, these findings should be interpreted with caution, as the reliability of both funnel plot interpretation and Egger's test is reduced with small study numbers (Figure 5).

### 3.5 Meta‐Analysis

**Figure 4:**
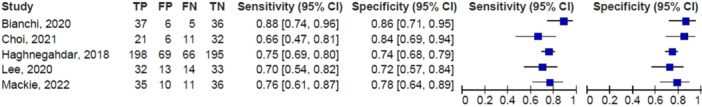
Forest plot showing the sensitivity and specificity.

In the meta‐analysis, five studies were included to estimate diagnostic performance. Observed diagnostic accuracy ranged from 0.66 to 0.88 across studies. Sensitivity values were generally moderate to high, ranging from 0.70 (95% CI 0.54–0.82) to 0.88 (95% CI 0.74–0.96), while specificity ranged from 0.72 (95% CI 0.57–0.84) to 0.86 (95% CI 0.71–0.95). As the small number of studies and methodological variability, these pooled estimates should be interpreted as descriptive summaries rather than precise or confirmatory measures of performance (Figure 4).

**Figure 5:**
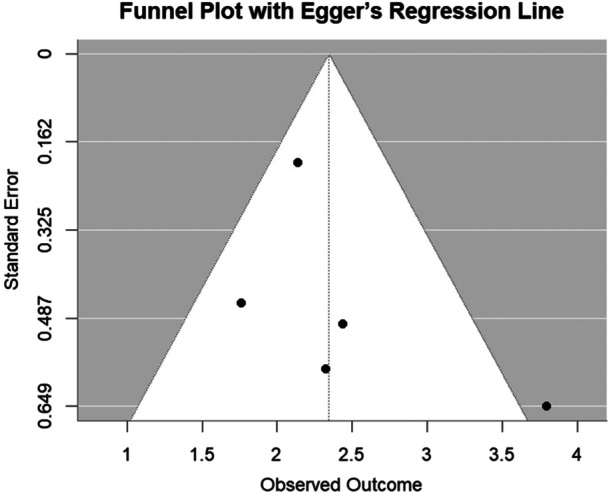
Funnel plot with Egger's regression line.

### 3.6 Sensitivity Analysis

**Figure 6:**
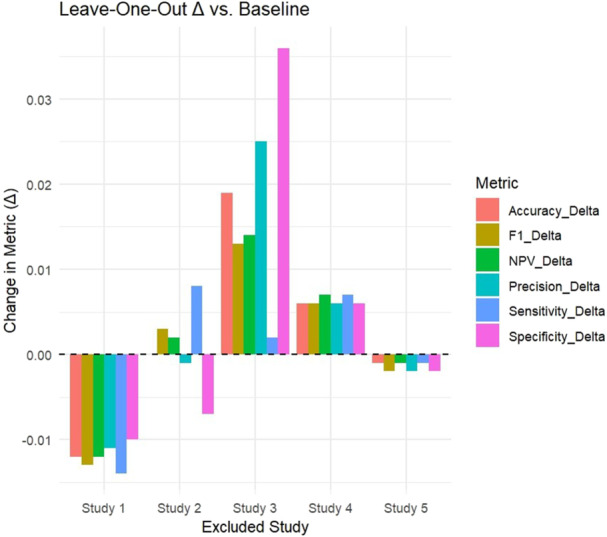
Sensitivity analysis.

When each study was excluded in turn, most metrics remained stable, with only minor changes (Δ < ±0.02), suggesting that pooled estimates were relatively stable across studies, although conclusions remain limited by the small sample size (Figure 6). Excluding Study 1 led to the largest reduction in sensitivity (Δ −0.014), accuracy (Δ −0.012), and F1 score (Δ −0.013), suggesting that this study contributed strongly to the stability of the pooled model. In contrast, exclusion of Study 3 resulted in notable increases in specificity (+0.036) and accuracy (+0.019), implying that this study exerted downward influence on specificity in the pooled analysis. Exclusion of Studies 2, 4, and 5 resulted in only minimal changes across all metrics (Δ ≤ ±0.01).

### 3.7 SROC

**Figure 7:**
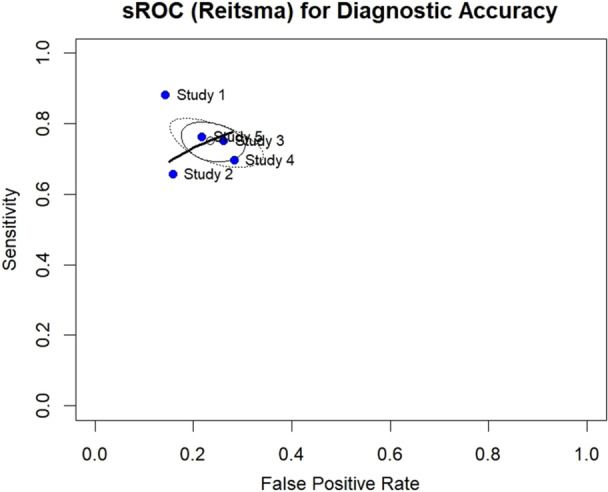
SROC.

The bivariate model estimated a pooled sensitivity of 0.751 (95% CI 0.703–0.794) and specificity of 0.766 (95% CI 0.711–0.814). The summary ROC curve yielded an AUC of 0.813, **suggesting moderate‐to‐good discriminatory ability**. Between‐study heterogeneity was modest (τ_sens = 0.089; τ_fpr = 0.161), with **an inverse relationship observed between sensitivity and specificity**. The sROC plot (Figure 7) showed study estimates clustering in the upper‐left quadrant, **indicating a general trend toward higher sensitivity with moderate specificity**, while the prediction ellipse illustrates expected variability in new populations.

These findings across analyses are best interpreted as exploratory and descriptive due to the limited number of included studies and substantial methodological heterogeneity.

## 4 Discussion

The present systematic review and meta‐analysis synthesized evidence from 14 studies evaluating artificial intelligence (AI)‐based methods against traditional approaches for the diagnosis and management of temporomandibular joint disorders (TMDs). AI models demonstrated moderate‐to‐high diagnostic accuracy, with pooled sensitivity between 0.70 and 0.88 and specificity between 0.72 and 0.86, consistent across included studies. These results underscore AI's potential as a valuable adjunct to established clinical and radiographic assessments.

### 4.1 Diagnostic Accuracy and Reliability

The diagnostic performance observed in our analysis aligns with earlier research highlighting AI's role in TMD diagnosis (Mehta et al. 2025). By restricting the meta‐analysis to compatible study designs, pooled estimates remained robust and interpretable. Sensitivity analyses showed that most metrics remained stable (Δ < ±0.02) when individual studies were excluded. Excluding Study 1 caused the largest reductions in sensitivity (Δ −0.014), accuracy (Δ −0.012), and F1 score (Δ −0.013), indicating its strong influence. Excluding Study 3 increased specificity (+0.036) and accuracy (+0.019), while exclusions of Studies 2, 4, and 5 had minimal effects (Δ ≤ ±0.01), supporting overall robustness. Sensitivity analyses confirmed the stability of these findings (Δ ≤ ±0.02), and the SROC curve further supported good discriminatory ability (AUC = 0.813; normalized pAUC = 0.729), despite moderate heterogeneity. Narrative synthesis of excluded studies ensured additional context without compromising pooled results. Funnel plot symmetry and Egger's test (*p* = 0.377) suggested no major asymmetry, though limited study numbers reduce reliability.

### 4.2 TMJ Subtypes and Imaging Modalities

Temporomandibular joint osteoarthritis (TMJOA) emerged as the most extensively investigated subtype, reflecting its clinical relevance in occlusion and aesthetics (de Dumast et al. 2018a; Barghan et al. 2012; Farook and Dudley 2023). Deep learning algorithms have been successfully applied to condylar morphology on CBCT images, with automated diagnostic tools developed using DC/TMD criteria (Lee et al. 2022). Our findings align with de Dumast, Mirabel, Paniagua et al. (2018), who reported 91% agreement between neural network classifiers and expert consensus in TMJOA detection. Both studies highlight the benefit of integrating multimodal imaging with clinical or biological markers, though current studies remain limited by small datasets and inconsistent validation.

### 4.3 Advancements in Imaging Approaches

Radiographic imaging remains central to TMJ evaluation. Barghan et al. (2012) emphasized CBCT's superiority over conventional radiography for high‐resolution assessment of osseous structures. Our review extends this conclusion by showing that AI applied to CBCT data further enhances precision (Barghan et al. 2012). Similarly, Kim et al. (2020) demonstrated that convolutional neural networks (CNNs) and R‐CNNs accurately detected mandibular condyles and classified osteoarthritis on panoramic radiographs, achieving high localization precision and reliable accuracy. These findings illustrate how AI can strengthen both advanced modalities, such as CBCT and more routine tools like panoramic imaging, reducing subjectivity in TMJ diagnostics (Kim et al. 2020).

### 4.4 Algorithmic Performance and Data Integration

AI algorithms varied in capability across studies. Gradient boosting models (XGBoost, LightGBM) performed strongly for structured datasets due to feature selection, computational efficiency, and reduced overfitting (Guan et al. 2023; Yi et al. 2023; Rufo et al. 2021; Mudawi 2024; Dalewski et al. 2019). Neural networks, particularly CNNs, excelled in unstructured imaging data tasks such as classification and segmentation (Jha et al. 2022; Diniz de Lima et al. 2022; Barghan et al. 2012; Farook and Dudley 2023; Jung et al. 2023). Support vector machines (SVMs) and k‐nearest neighbors (KNN) offered reliable pattern recognition, while natural language processing (NLP) extracted structured information from free‐text records (Xu et al. 2023; Ribera et al. 2019; de Dumast, Mirabel, Paniagua et al. 2018; Zhang et al. 2021). Combining semantic (pain, symptoms) with radiomic (imaging features) data consistently improved diagnostic performance (Jha et al. 2022). Our findings are consistent with Farook and Dudley (2023), who noted strong accuracy for TMJOA detection but poorer results for disc disorders using MRI, largely due to non‐standardized datasets and imaging protocols (Farook and Dudley 2023). These results highlight the need for harmonized acquisition standards and external validation before AI adoption in routine care. Although several studies employed deep learning architectures, observed performance differences between machine learning and deep learning approaches were inconsistent and appeared task‐ and modality‐dependent; therefore, no definitive conclusions regarding model superiority can be drawn.

Across the TMJ/TMD AI literature, systematic and umbrella reviews have highlighted that explainability is often reported (e.g., via saliency maps or feature‐importance outputs) but is less frequently evaluated in terms of clinical utility, such as whether it improves clinician understanding or decision‐making (Jha et al. 2022; Mehta et al. 2025; Farook and Dudley 2023). Similarly, external validation and broader assessments of generalizability remain limited in many published TMJ/TMD AI studies, which restricts confidence in real‐world performance across settings and populations (Jha et al. 2022; Almășan et al. 2023). These recurring limitations likely reflect small datasets, predominantly single‐center designs, and inconsistent reporting standards, reinforcing that reported transparency does not necessarily equate to demonstrated trustworthiness (Whiting et al. 2011; Mehta et al. 2025).

### 4.5 Clinical Implications and Workflow Efficiency

Beyond diagnostic accuracy, AI has the potential to streamline clinical workflows. Lee et al. (2022) showed that deep learning‐based integration of intraoral scans with CBCT achieved comparable accuracy to manual methods but with greater efficiency (Lee et al. 2022). Likewise, Tran et al. (2022) also emphasized that while conservative‐first approaches to TMD remain standard, traditional methods often lack personalization and early diagnostic precision. AI can complement these strategies by enabling earlier detection, patient‐specific classification, and more efficient decision‐making, thereby enhancing reproducibility and reducing variability (Tran et al. 2022). Our study reinforces the growing role of AI in enhancing diagnostic accuracy and clinical efficiency in TMD management.

### 4.6 Alignment With Clinical Guidelines and Future Directions

In view of this, the recently developed Diagnostic Criteria for Temporomandibular Disorders (DC/TMD) protocol offers an evidence‐based framework that can serve as a guiding template for both clinical practice and research (Schiffman et al. 2014). This standardized approach supports the full range of diagnostic activities from screening to definitive evaluation and fosters a common language among clinicians worldwide. Importantly, the DC/TMD protocol's emphasis on integrating clinical, psychosocial, and imaging data aligns well with emerging AI methodologies, underscoring the potential for synergy between traditional diagnostic frameworks and novel AI tools. Ultimately, adapting such guidelines to diverse cultural, social, educational, and healthcare contexts will be essential for optimizing TMD management on both national and international levels. These advancements align with the 2024 INfORM/IADR consensus, which emphasizes a patient‐centered, biopsychosocial model grounded in validated assessment and conservative care. The guidelines caution against irreversible treatments and unnecessary imaging, promoting evidence‐based decision‐making. Integrating AI within this framework can support more personalized, guideline‐compliant clinical practice (Manfredini et al. 2025).

From an implementation‐science perspective, future studies should assess how AI tools influence real‐world clinical decision‐making rather than model accuracy alone. A pragmatic research question is whether incorporating explainability features (e.g., Grad‐CAM heatmaps) reduces automation bias and improves diagnostic agreement between dental students and experienced clinicians during complex radiographic interpretation. Such work would inform clinician trust, usability, and safe integration of AI into routine TMD workflows.

### 4.7 Limitations

Despite encouraging results, current AI research in temporomandibular disorders (TMD) remains constrained by methodological weaknesses. Most studies involved small sample sizes (mean *n* = 42), lacked blinding or pre‐specified diagnostic thresholds, and carried a high risk of bias. External validation was rarely conducted, limiting generalizability, and publication bias cannot be excluded given the small number of available studies. Direct head‐to‐head comparisons between AI‐assisted and traditional diagnostic methods were also scarce. Study quality varied as several lacked blinding or pre‐specified thresholds, affecting internal validity. The substantial methodological diversity across included studies limits direct comparability; therefore, findings should be interpreted as descriptive trends rather than definitive performance comparisons. Future research should prioritize multicenter, prospective studies with standardized reporting, larger and more diverse datasets, and transparent AI development pipelines. Incorporating multimodal inputs, including imaging, clinical, and biological markers, represents a promising strategy to improve diagnostic accuracy and personalized treatment planning.

## 5 Conclusion

AI algorithms, particularly those using machine learning and radiomic feature analysis, show emerging promise for supporting decision‐making in temporomandibular disorders (TMD). While traditional methods remain the gold standard, AI may augment existing frameworks; however, current evidence is preliminary and heterogeneous. The available literature is constrained by small sample sizes, high risk of bias, inconsistent reporting, and limited external validation, which restrict direct comparability and preclude definitive conclusions regarding clinical superiority. Accordingly, this review primarily identifies important methodological and reporting gaps rather than establishing effectiveness. Future research should prioritize well‐designed, multicenter validation studies with standardized protocols, transparent AI development pipelines, and high‐quality open datasets. For clinical translation, careful evaluation across diverse populations and integration within established DC/TMD workflows will be necessary before routine implementation.

## Author Contributions

Conceptualization: Vini Mehta. Data acquisition and analysis: Annie Vathani, Praveen Kumar Gonuguntla Kamma, Ankita Mathur, and Saurabh Chaturvedi. Resources: Vini Mehta and Toufiq Noor. Manuscript Writing Original: Vini Mehta, Annie Vathani, and Praveen Kumar Gonuguntla Kamma. Manuscript review and editing: Saurabh Chaturvedi, Ankita Mathur, and Toufiq Noor.

## Funding

The authors have nothing to report.

## Consent

The authors have nothing to report.

## Conflicts of Interest

The authors declare no conflicts of interest.

## Supporting information

Supporting File:

## Data Availability

The data that supports the findings of this study are available in the supplementary information of this article.
