# Supplementary material for: Comparing Traditional Versus AI‐Assisted TMJ Disorder Management Approaches: A Systematic Review and Meta‐Analysis
Source: Clin Exp Dent Res. 2026 Apr 28;12(2):e70370. doi: 10.1002/cre2.70370 (PMC13122102; doi:10.1002/cre2.70370)
Supplement: Supplementary file 1 — Supporting File: [file CRE2-12-e70370-s001.docx]

**SUPPLEMENTARY SHEET**

**Supplementary Table 1: PRISMA Checklist**

| **Section and Topic** | **Item #** | **Checklist item** | **Location where item is reported (Line Number mentioned)** |
| --- | --- | --- | --- |
| **TITLE** | | |  |
| Title | 1 | Identify the report as a systematic review. | 2 |
| **ABSTRACT** | | |  |
| Abstract | 2 | See the PRISMA 2020 for Abstracts checklist. | 8 |
| **INTRODUCTION** | | |  |
| Rationale | 3 | Describe the rationale for the review in the context of existing knowledge. | 69-84 |
| Objectives | 4 | Provide an explicit statement of the objective(s) or question(s) the review addresses. | 89-90 |
| **METHODS** | | |  |
| Eligibility criteria | 5 | Specify the inclusion and exclusion criteria for the review and how studies were grouped for the syntheses. | 109-121 |
| Information sources | 6 | Specify all databases, registers, websites, organisations, reference lists and other sources searched or consulted to identify studies. Specify the date when each source was last searched or consulted. | 103-105 |
| Search strategy | 7 | Present the full search strategies for all databases, registers and websites, including any filters and limits used. | 102-107 |
| Selection process | 8 | Specify the methods used to decide whether a study met the inclusion criteria of the review, including how many reviewers screened each record and each report retrieved, whether they worked independently, and if applicable, details of automation tools used in the process. | 123-125 |
| Data collection process | 9 | Specify the methods used to collect data from reports, including how many reviewers collected data from each report, whether they worked independently, any processes for obtaining or confirming data from study investigators, and if applicable, details of automation tools used in the process. | 123-130 |
| Data items | 10a | List and define all outcomes for which data were sought. Specify whether all results that were compatible with each outcome domain in each study were sought (e.g. for all measures, time points, analyses), and if not, the methods used to decide which results to collect. | 127-130 |
|  | 10b | List and define all other variables for which data were sought (e.g. participant and intervention characteristics, funding sources). Describe any assumptions made about any missing or unclear information. | 130-132 |
| Study risk of bias assessment | 11 | Specify the methods used to assess risk of bias in the included studies, including details of the tool(s) used, how many reviewers assessed each study and whether they worked independently, and if applicable, details of automation tools used in the process. | 133-143 |
| Effect measures | 12 | Specify for each outcome the effect measure(s) (e.g. risk ratio, mean difference) used in the synthesis or presentation of results. | 145 |
| Synthesis methods | 13a | Describe the processes used to decide which studies were eligible for each synthesis (e.g. tabulating the study intervention characteristics and comparing against the planned groups for each synthesis (item #5)). | - |
|  | 13b | Describe any methods required to prepare the data for presentation or synthesis, such as handling of missing summary statistics, or data conversions. | 106-108 |
|  | 13c | Describe any methods used to tabulate or visually display results of individual studies and syntheses. | 145-147 |
|  | 13d | Describe any methods used to synthesize results and provide a rationale for the choice(s). If meta-analysis was performed, describe the model(s), method(s) to identify the presence and extent of statistical heterogeneity, and software package(s) used. | 145-151 |
|  | 13e | Describe any methods used to explore possible causes of heterogeneity among study results (e.g. subgroup analysis, meta-regression). | 151 |
|  | 13f | Describe any sensitivity analyses conducted to assess robustness of the synthesized results. | - |
| Reporting bias assessment | 14 | Describe any methods used to assess risk of bias due to missing results in a synthesis (arising from reporting biases). | - |
| Certainty assessment | 15 | Describe any methods used to assess certainty (or confidence) in the body of evidence for an outcome. | 150-151 |
| **RESULTS** | | |  |
| Study selection | 16a | Describe the results of the search and selection process, from the number of records identified in the search to the number of studies included in the review, ideally using a flow diagram. | 162-163 |
|  | 16b | Cite studies that might appear to meet the inclusion criteria, but which were excluded, and explain why they were excluded. | - |
| Study characteristics | 17 | Cite each included study and present its characteristics. | 167-168 |
| Risk of bias in studies | 18 | Present assessments of risk of bias for each included study. | 182-183 |
| Results of individual studies | 19 | For all outcomes, present, for each study: (a) summary statistics for each group (where appropriate) and (b) an effect estimate and its precision (e.g. confidence/credible interval), ideally using structured tables or plots. | 184 |
| Results of syntheses | 20a | For each synthesis, briefly summarise the characteristics and risk of bias among contributing studies. | 193-195 |
|  | 20b | Present results of all statistical syntheses conducted. If meta-analysis was done, present for each the summary estimate and its precision (e.g. confidence/credible interval) and measures of statistical heterogeneity. If comparing groups, describe the direction of the effect. | 216 |
|  | 20c | Present results of all investigations of possible causes of heterogeneity among study results. | 216-222 |
|  | 20d | Present results of all sensitivity analyses conducted to assess the robustness of the synthesized results. | 222 |
| Reporting biases | 21 | Present assessments of risk of bias due to missing results (arising from reporting biases) for each synthesis assessed. | - |
| Certainty of evidence | 22 | Present assessments of certainty (or confidence) in the body of evidence for each outcome assessed. | - |
| **DISCUSSION** | | |  |
| Discussion | 23a | Provide a general interpretation of the results in the context of other evidence. | 226 |
|  | 23b | Discuss any limitations of the evidence included in the review. | 277 |
|  | 23c | Discuss any limitations of the review processes used. | 280 |
|  | 23d | Discuss implications of the results for practice, policy, and future research. | 293 |
| **OTHER INFORMATION** | | |  |
| Registration and protocol | 24a | Provide registration information for the review, including register name and registration number, or state that the review was not registered. | 84 |
|  | 24b | Indicate where the review protocol can be accessed, or state that a protocol was not prepared. | - |
|  | 24c | Describe and explain any amendments to information provided at registration or in the protocol. | 84 |
| Support | 25 | Describe sources of financial or non-financial support for the review, and the role of the funders or sponsors in the review. | 382 |
| Competing interests | 26 | Declare any competing interests of review authors. | 380 |
| Availability of data, code and other materials | 27 | Report which of the following are publicly available and where they can be found: template data collection forms; data extracted from included studies; data used for all analyses; analytic code; any other materials used in the review. |  |

*From:*  Page MJ, McKenzie JE, Bossuyt PM, Boutron I, Hoffmann TC, Mulrow CD, et al. The PRISMA 2020 statement: an updated guideline for reporting systematic reviews. BMJ 2021;372:n71. doi: 10.1136/bmj.n71

**Supplementary Table 2: Search Strategy**

| Data Base | Search Query | No.of Articles |
| --- | --- | --- |
| PubMed | ("artificial intelligence " OR " neural network " OR " machine learning " OR " deep learning ")) AND/OR (("TMJ osteoarthritis" OR "Temporomandibular joint osteoarthritis" OR " Temporomandibular disorders " OR "TMDs" OR "TMJ disorder" OR "Temporomandibular joint disorders" OR "TMJ arthritis" OR "Temporomandibular joint arthritis" OR "masticatory muscle disorder" OR "degenerative joint disease" OR "Temporomandibular joint disease" OR "TMJ disease" ) AND ("Management" OR "Therpahy" OR "treatment") | 746 |
| Embase | ('artificial intelligence'/exp OR 'artificial intelligence' OR on 'neural network'/exp OR 'neural network' OR 'machine learning'/exp OR 'machine learning' OR 'deep learning'/exp OR 'deep learning')  AND  ('temporomandibular joint disease' OR 'tmj disease' OR 'temporomandibular joint osteoarthritis'/exp OR 'temporomandibular joint osteoarthritis' OR 'tmj osteoarthritis' OR through 'temporomandibular disorders'/exp OR 'temporomandibular disorders' OR 'tmds' OR 'tmj disorder' OR 'temporomandibular joint disorders' OR 'tmj arthritis' OR 'temporomandibular joint arthritis' OR 'masticatory muscle disorder' OR 'degenerative joint disease'/exp OR 'degenerative joint disease') |  |

**Supplementary Table 3: List of excluded studies**

| **Reasons**  **for exclusion** | **Number of studies** | **List of excluded articles** |
| --- | --- | --- |
| Studies related to digital therapeutics as a diagnosis | 1 | Park SY, Byun SH, Yang BE, Kim D, Kim B, Lee JH, Kim YK. Randomized controlled trial of digital therapeutics for temporomandibular disorder: A pilot study. J Dent. 2024 Aug;147:105030. doi: 10.1016/j.jdent.2024.105030. Epub 2024 Apr 27. PMID: 38685341. |
| Studies does not focus on Temporomandibular joint (TMJ) disorders in the context of diagnosis or management | 2 | Kraaijenga S, van der Molen L, van Tinteren H, Hilgers F, Smeele L. Treatment of myogenic temporomandibular disorder: a prospective randomized clinical trial, comparing a mechanical stretching device (TheraBite®) with standard physical therapy exercise. Cranio. 2014 Jul;32(3):208-16. doi: 10.1179/0886963413Z.00000000016. Epub 2014 Jan 24. PMID: 25000163. |
|  |  | Ariji Y, Nakayama M, Nishiyama W, Ogi N, Sakuma S, Katsumata A, Kurita K, Ariji E. Potential clinical application of masseter and temporal muscle massage treatment using an oral rehabilitation robot in temporomandibular disorder patients with myofascial pain. Cranio. 2015 Oct;33(4):256-62. doi: 10.1080/08869634.2015.1097303. Epub 2015 Dec 29. PMID: 26714800. |
| Studies related to AI diagnostic imaging with no web repository storage | 5 | Barghan S, Tetradis S, Mallya S. Application of cone beam computed tomography for assessment of the temporomandibular joints. Aust Dent J. 2012 Mar;57 Suppl 1:109-18. doi: 10.1111/j.1834-7819.2011.01663.x. PMID: 22376103. |
|  |  | Yatabe M, Prieto JC, Styner M, Zhu H, Ruellas AC, Paniagua B, Budin F, Benavides E, Shoukri B, Michoud L, Ribera N, Cevidanes L. 3D superimposition of craniofacial imaging-The utility of multicentre collaborations. Orthod Craniofac Res. 2019 May;22 Suppl 1(Suppl 1):213-220. doi: 10.1111/ocr.12281. PMID: 31074129; PMCID: PMC6660909. |
|  |  | Ferraz AM Jr, Devito KL, Guimarães JP. Temporomandibular disorder in patients with juvenile idiopathic arthritis: clinical evaluation and correlation with the findings of cone beam computed tomography. Oral Surg Oral Med Oral Pathol Oral Radiol. 2012 Sep;114(3):e51-7. doi: 10.1016/j.oooo.2012.02.010. Epub 2012 Jul 6. PMID: 22771410. |
|  |  | Paniagua B, Cevidanes L, Walker D, Zhu H, Guo R, Styner M. Clinical application of SPHARM-PDM to quantify temporomandibular joint osteoarthritis. Comput Med Imaging Graph. 2011 Jul;35(5):345-52. doi: 10.1016/j.compmedimag.2010.11.012. Epub 2010 Dec 24. PMID: 21185694; PMCID: PMC3083466. |
|  |  | Michoud L, Huang C, Yatabe M, Ruellas A, Ioshida M, Paniagua B, Styner M, Gonçalves JR, Bianchi J, Cevidanes L, Prieto JC. A web-based system for statistical shape analysis in temporomandibular joint osteoarthritis. Proc SPIE Int Soc Opt Eng. 2019 Feb;10953:109530T. doi: 10.1117/12.2506032. Epub 2019 Mar 15. PMID: 31057201; PMCID: PMC6494085. |
| Studies with no focus on AI approaches but other means of digital forms. | 6 | Michoud L, Huang C, Yatabe M, Ruellas A, Ioshida M, Paniagua B, Styner M, Gonçalves JR, Bianchi J, Cevidanes L, Prieto JC. A web-based system for statistical shape analysis in temporomandibular joint osteoarthritis. Proc SPIE Int Soc Opt Eng. 2019 Feb;10953:109530T. doi: 10.1117/12.2506032. Epub 2019 Mar 15. PMID: 31057201; PMCID: PMC6494085. |
|  |  | Chen CC, Lin CC, Hsieh HP, Fu YC, Chen YJ, Lu TW. In vivo three-dimensional mandibular kinematics and functional point trajectories during temporomandibular activities using 3d fluoroscopy. Dentomaxillofac Radiol. 2021 Feb 1;50(2):20190464. doi: 10.1259/dmfr.20190464. Epub 2020 Aug 12. PMID: 32783637; PMCID: PMC7860955. |
|  |  | Shu J, Ma H, Xiong X, Shao B, Zheng T, Liu Y, Liu Z. Mathematical analysis of the condylar trajectories in asymptomatic subjects during mandibular motions. Med Biol Eng Comput. 2021 Apr;59(4):901-911. doi: 10.1007/s11517-021-02346-6. Epub 2021 Mar 28. PMID: 33774756. |
|  |  | Leissner O, Maulén-Yáñez M, Meeder-Bella W, León-Morales C, Vergara-Bruna E, González-Arriagada WA. Assessment of mandibular kinematics values and its relevance for the diagnosis of temporomandibular joint disorders. J Dent Sci. 2021 Jan;16(1):241-248. doi: 10.1016/j.jds.2020.05.015. Epub 2020 Jun 6. PMID: 33384804; PMCID: PMC7770294. |
|  |  | Iwase Y, Sugiki T, Kise Y, Nishiyama M, Nozawa M, Fukuda M, Ariji Y, Ariji E. Deep learning classification performance for diagnosing condylar osteoarthritis in patients with dentofacial deformities using panoramic temporomandibular joint projection images. Oral Radiol. 2024 Oct;40(4):538-545. doi: 10.1007/s11282-024-00768-0. Epub 2024 Jul 11. PMID: 38990220. |
|  |  | Baltali E, Zhao KD, Koff MF, Durmuş E, An KN, Keller EE. A method for quantifying condylar motion in patients with osteoarthritis using an electromagnetic tracking device and computed tomography imaging. J Oral Maxillofac Surg. 2008 May;66(5):848-57. doi: 10.1016/j.joms.2008.01.021. PMID: 18423270. |
